# 6-(2-Fluoro­phen­yl)-5,6-dihydro­benzimidazolo[1,2-*c*]quinazoline

**DOI:** 10.1107/S1600536808031875

**Published:** 2008-10-11

**Authors:** Ai-Ke Li, Jian-Xin Chen, Li-Qun Zheng, Mei-Ping Zhu, Li Zhang

**Affiliations:** a College of Chemistry and Materials Science, Fujian Normal University, Fuzhou, Fujian 350007, People’s Republic of China

## Abstract

In the title compound, C_20_H_14_FN_3_, the pyrimidine ring adopts a half-chair conformation. The dihedral angle between the benzimidazole ring system and the fluoro­phenyl ring is 84.18 (10)°. In the crystal structure, mol­ecules are linked into a two-dimensional network parallel to the *bc* plane by N—H⋯N and C—H⋯F hydrogen bonds.

## Related literature

For related structures, see: Elgemeie *et al.* (1998 ▶); Jayalakshmi *et al.* (2004 ▶); Low *et al.* (2003 ▶); Mahendra *et al.* (2005 ▶). For related literature, see: Alexandre *et al.* (2003 ▶); Bandurco *et al.* (1981 ▶); Chern *et al.* (1993 ▶); Fatmi *et al.* (1984 ▶).
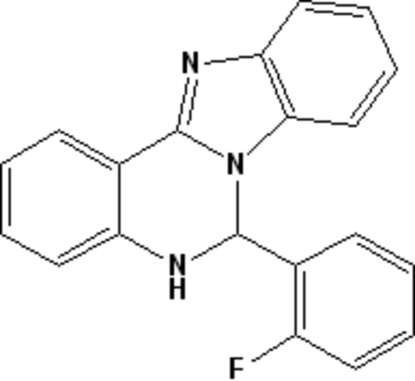


## Experimental

### 

#### Crystal data

C_20_H_14_FN_3_
*M* *_r_* = 315.34
Monoclinic, 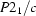

*a* = 8.7344 (17) Å
*b* = 13.623 (3) Å
*c* = 13.356 (3) Å
β = 99.78 (3)°
*V* = 1566.1 (6) Å^3^
*Z* = 4
Mo *K*α radiation
μ = 0.09 mm^−1^
*T* = 298 (2) K
0.23 × 0.21 × 0.15 mm

#### Data collection

Rigaku R-AXIS RAPID IP diffractometer
Absorption correction: multi-scan (*RAPID-AUTO*; Rigaku, 1998 ▶) *T*_min_ = 0.899, *T*_max_ = 0.991
15171 measured reflections
3591 independent reflections
2286 reflections with *I* > 2σ(*I*)
*R*_int_ = 0.035

#### Refinement

*R*[*F* ^2^ > 2σ(*F* ^2^)] = 0.054
*wR*(*F* ^2^) = 0.160
*S* = 1.06
3591 reflections
217 parameters
H-atom parameters constrained
Δρ_max_ = 0.33 e Å^−3^
Δρ_min_ = −0.31 e Å^−3^

### 

Data collection: *RAPID-AUTO* (Rigaku, 1998 ▶); cell refinement: *RAPID-AUTO*; data reduction: *RAPID-AUTO*; program(s) used to solve structure: *SHELXS97* (Sheldrick, 2008 ▶); program(s) used to refine structure: *SHELXL97* (Sheldrick, 2008 ▶); molecular graphics: *SHELXTL/PC* (Sheldrick, 2008 ▶); software used to prepare material for publication: *SHELXL97*.

## Supplementary Material

Crystal structure: contains datablocks I, global. DOI: 10.1107/S1600536808031875/ci2668sup1.cif

Structure factors: contains datablocks I. DOI: 10.1107/S1600536808031875/ci2668Isup2.hkl

Additional supplementary materials:  crystallographic information; 3D view; checkCIF report

## Figures and Tables

**Table 1:** Hydrogen-bond geometry (Å, °)

*D*—H⋯*A*	*D*—H	H⋯*A*	*D*⋯*A*	*D*—H⋯*A*
N1—H1*A*⋯N3^i^	0.86	2.31	2.995 (2)	136
C11—H11*A*⋯F1^ii^	0.93	2.43	3.264 (3)	149

Symmetry codes: (i) 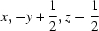; (ii) 
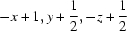
.

**Fig. 1.**
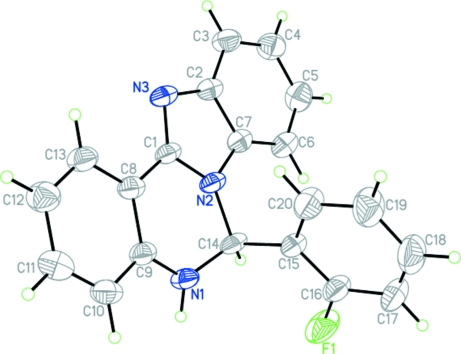
The molecular structure of the title compound, showing the atom-numbering scheme. Displacement ellipsoids are drawn at the 50% probability level.

**Fig. 2.**
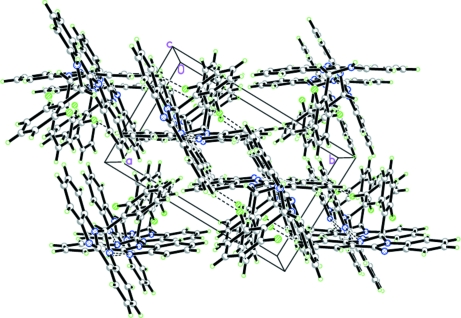
The crystal packing of the title compound, viewed along the c axis. Hydrogen bonds are shown as dashed lines.

## supplementary crystallographic information

### Comment

A variety of compounds containing the quinazoline skeleton has been found to exhibit antihypertensive, antimalarial and bronchodilator activities (Alexandre *et al.*, 2003; Bandurco *et al.*, 1981; Chern *et al.*, 1993; Fatmi *et al.*, 1984) and crystal structures of some of these compounds have been reported (Elgemeie *et al.*, 1998; Low *et al.*, 2003; Jayalakshmi *et al.*, 2004; Mahendra *et al.*, 2005). In view of the above importance, the title compound, (I), was prepared from ο-aminophenylbenzimidazole and ο-fluorobenzaldehyde and its crystal structure is reported here (Fig. 1).

Most of the bond lengths and angles have normal values and are comparable to those observed in related structures (Low *et al.*, 2003; Mahendra *et al.*, 2005). The benzimidazole ring system is planar. The pyrimidine ring adopts a half-chair conformation, with atoms N1 and C14 deviate from the N2/C1/C8/C9 plane by 0.178 (3) and -0.222 (3) Å, respectively. The dihedral angle between the benzimidazole ring system and the fluorophenyl ring is 84.18 (10)°.

In the crystal structure, the molecules are linked into a two-dimensional network parallel to the *bc* plane (Fig.2) by N—H···N and C—H···F hydrogen bonds (Table 1).

### Experimental

All reagents were of AR grade available commercially and used without further purification. A solution of ο-aminophenylbenzimidazole (5 mmol) and ο-fluorobenzaldehyde (5 mmol) in ethanol (12 ml) was treated with acetic acid (0.2 ml) for 5 h. The resulting solution was concentrated under reduced pressure to a small volume to obtain a creamy compound. The solid was recrystallized from ethanol to give a brown crystalline compound (I) (yield 70%; m.p. 521 K). Single crystals suitable for X-ray diffraction analysis were obtained by slow evaporation of an ethanol solution.

### Refinement

All H atoms were placed in calculated positions with N—H = 0.86 Å and C—H = 0.93–0.98 Å, and refined using a riding model with *U*_iso_(H) = 1.2*U*_eq_(C,*N*).

### Crystal data

**Table:**

C_20_H_14_FN_3_	*F*(000) = 656
*M**_r_* = 315.34	*D*_x_ = 1.337 Mg m^−^^3^
Monoclinic, *P*2_1_/*c*	Mo *K*α radiation, λ = 0.71073 Å
Hall symbol: -P 2ybc	Cell parameters from 3589 reflections
*a* = 8.7344 (17) Å	θ = 3.0–27.5°
*b* = 13.623 (3) Å	µ = 0.09 mm^−^^1^
*c* = 13.356 (3) Å	*T* = 298 K
β = 99.78 (3)°	Chunk, brown
*V* = 1566.1 (6) Å^3^	0.23 × 0.21 × 0.15 mm
*Z* = 4	

### Data collection

**Table:**

Rigaku Weissenberg IP diffractometer	3591 independent reflections
Radiation source: sealed tube	2286 reflections with *I* > 2σ(*I*)
graphite	*R*_int_ = 0.035
φ and ω scans	θ_max_ = 27.5°, θ_min_ = 3.0°
Absorption correction: multi-scan (RAPID-AUTO; Rigaku, 1998)	*h* = −11→11
*T*_min_ = 0.899, *T*_max_ = 0.991	*k* = −17→16
15171 measured reflections	*l* = −17→17

### Refinement

**Table:**

Refinement on *F*^2^	Primary atom site location: structure-invariant direct methods
Least-squares matrix: full	Secondary atom site location: difference Fourier map
*R*[*F*^2^ > 2σ(*F*^2^)] = 0.054	Hydrogen site location: inferred from neighbouring sites
*wR*(*F*^2^) = 0.160	H-atom parameters constrained
*S* = 1.06	*w* = 1/[σ^2^(*F*_o_^2^) + (0.0751*P*)^2^ + 0.2459*P*] where *P* = (*F*_o_^2^ + 2*F*_c_^2^)/3
3591 reflections	(Δ/σ)_max_ = 0.001
217 parameters	Δρ_max_ = 0.33 e Å^−^^3^
0 restraints	Δρ_min_ = −0.31 e Å^−^^3^

### Special details

**Table:**

Geometry. All e.s.d.'s (except the e.s.d. in the dihedral angle between two l.s. planes) are estimated using the full covariance matrix. The cell e.s.d.'s are taken into account individually in the estimation of e.s.d.'s in distances, angles and torsion angles; correlations between e.s.d.'s in cell parameters are only used when they are defined by crystal symmetry. An approximate (isotropic) treatment of cell e.s.d.'s is used for estimating e.s.d.'s involving l.s. planes.
Refinement. Refinement of *F*^2^ against ALL reflections. The weighted *R*-factor *wR* and goodness of fit *S* are based on *F*^2^, conventional *R*-factors *R* are based on *F*, with *F* set to zero for negative *F*^2^. The threshold expression of *F*^2^ > σ(*F*^2^) is used only for calculating *R*-factors(gt) *etc*. and is not relevant to the choice of reflections for refinement. *R*-factors based on *F*^2^ are statistically about twice as large as those based on *F*, and *R*- factors based on ALL data will be even larger.

### Fractional atomic coordinates and isotropic or equivalent isotropic displacement parameters (Å^2^)

**Table:**

	*x*	*y*	*z*	*U*_iso_*/*U*_eq_	
F1	0.22484 (19)	0.16127 (14)	0.15884 (10)	0.1072 (6)	
N1	0.51477 (19)	0.25468 (13)	0.34079 (11)	0.0606 (5)	
H1A	0.5498	0.2553	0.2844	0.073*	
N2	0.41799 (18)	0.15511 (12)	0.45924 (10)	0.0519 (4)	
N3	0.4822 (2)	0.16940 (12)	0.62780 (11)	0.0571 (4)	
C1	0.4941 (2)	0.20758 (14)	0.53935 (12)	0.0500 (4)	
C2	0.3860 (2)	0.08770 (15)	0.60402 (14)	0.0553 (5)	
C3	0.3286 (3)	0.02189 (17)	0.66787 (16)	0.0698 (6)	
H3B	0.3564	0.0268	0.7381	0.084*	
C4	0.2299 (3)	−0.05068 (18)	0.62477 (19)	0.0769 (6)	
H4A	0.1894	−0.0949	0.6664	0.092*	
C5	0.1893 (3)	−0.05921 (18)	0.5196 (2)	0.0793 (7)	
H5A	0.1232	−0.1095	0.4924	0.095*	
C6	0.2453 (3)	0.00545 (16)	0.45505 (17)	0.0671 (6)	
H6A	0.2180	0.0003	0.3848	0.081*	
C7	0.3436 (2)	0.07804 (14)	0.49963 (14)	0.0541 (5)	
C8	0.5723 (2)	0.29641 (14)	0.51872 (13)	0.0517 (4)	
C9	0.5753 (2)	0.31982 (15)	0.41612 (13)	0.0523 (5)	
C10	0.6471 (3)	0.40634 (17)	0.39484 (16)	0.0646 (6)	
H10A	0.6476	0.4239	0.3276	0.077*	
C11	0.7168 (3)	0.46590 (18)	0.47110 (19)	0.0747 (6)	
H11A	0.7660	0.5230	0.4551	0.090*	
C12	0.7158 (3)	0.44306 (17)	0.57132 (18)	0.0736 (6)	
H12A	0.7646	0.4842	0.6225	0.088*	
C13	0.6429 (3)	0.35968 (15)	0.59553 (15)	0.0621 (5)	
H13A	0.6401	0.3450	0.6632	0.075*	
C14	0.3936 (2)	0.18490 (15)	0.35345 (12)	0.0528 (5)	
H14A	0.4050	0.1270	0.3119	0.063*	
C15	0.2318 (2)	0.22592 (14)	0.32082 (13)	0.0522 (5)	
C16	0.1535 (3)	0.21340 (17)	0.22401 (16)	0.0668 (6)	
C17	0.0088 (3)	0.2507 (2)	0.1890 (2)	0.0894 (8)	
H17A	−0.0400	0.2398	0.1224	0.107*	
C18	−0.0614 (3)	0.3038 (2)	0.2538 (3)	0.0944 (8)	
H18A	−0.1598	0.3297	0.2317	0.113*	
C19	0.0113 (3)	0.3198 (2)	0.3516 (2)	0.0937 (8)	
H19A	−0.0373	0.3570	0.3955	0.112*	
C20	0.1567 (3)	0.28070 (19)	0.38496 (18)	0.0762 (7)	
H20A	0.2051	0.2913	0.4517	0.091*	

### Atomic displacement parameters (Å^2^)

**Table:**

	*U*^11^	*U*^22^	*U*^33^	*U*^12^	*U*^13^	*U*^23^
F1	0.1146 (12)	0.1504 (14)	0.0498 (8)	0.0203 (11)	−0.0054 (7)	−0.0329 (8)
N1	0.0651 (10)	0.0853 (12)	0.0328 (7)	0.0036 (9)	0.0128 (7)	0.0029 (7)
N2	0.0612 (9)	0.0631 (9)	0.0304 (7)	0.0075 (8)	0.0047 (6)	0.0008 (6)
N3	0.0700 (10)	0.0691 (10)	0.0320 (8)	0.0019 (9)	0.0081 (7)	0.0012 (7)
C1	0.0579 (10)	0.0610 (11)	0.0305 (8)	0.0078 (9)	0.0056 (7)	−0.0013 (7)
C2	0.0646 (12)	0.0598 (11)	0.0414 (10)	0.0055 (10)	0.0088 (8)	0.0028 (8)
C3	0.0853 (15)	0.0752 (14)	0.0496 (11)	−0.0042 (12)	0.0131 (10)	0.0088 (10)
C4	0.0859 (16)	0.0714 (14)	0.0734 (15)	−0.0053 (13)	0.0136 (12)	0.0156 (12)
C5	0.0819 (16)	0.0672 (13)	0.0852 (18)	−0.0053 (12)	0.0033 (13)	−0.0018 (13)
C6	0.0759 (14)	0.0694 (13)	0.0521 (11)	0.0037 (11)	−0.0002 (10)	−0.0035 (10)
C7	0.0595 (11)	0.0560 (10)	0.0456 (10)	0.0079 (9)	0.0057 (8)	0.0000 (8)
C8	0.0566 (10)	0.0591 (11)	0.0406 (9)	0.0083 (9)	0.0113 (8)	0.0024 (8)
C9	0.0507 (10)	0.0679 (12)	0.0391 (9)	0.0131 (9)	0.0100 (8)	0.0030 (8)
C10	0.0688 (13)	0.0753 (13)	0.0530 (12)	0.0096 (11)	0.0205 (10)	0.0159 (10)
C11	0.0840 (16)	0.0704 (14)	0.0751 (15)	−0.0008 (12)	0.0290 (13)	0.0086 (12)
C12	0.0897 (16)	0.0698 (13)	0.0639 (14)	−0.0097 (13)	0.0202 (12)	−0.0090 (11)
C13	0.0754 (13)	0.0693 (13)	0.0427 (10)	−0.0020 (11)	0.0131 (9)	−0.0044 (9)
C14	0.0653 (11)	0.0648 (11)	0.0270 (8)	0.0133 (10)	0.0041 (7)	−0.0026 (8)
C15	0.0587 (11)	0.0567 (10)	0.0392 (9)	0.0054 (9)	0.0026 (8)	−0.0002 (8)
C16	0.0716 (14)	0.0800 (14)	0.0444 (10)	0.0017 (11)	−0.0027 (9)	−0.0029 (10)
C17	0.0748 (16)	0.114 (2)	0.0674 (15)	0.0012 (15)	−0.0206 (13)	0.0111 (15)
C18	0.0635 (15)	0.103 (2)	0.110 (2)	0.0173 (15)	−0.0057 (15)	0.0153 (18)
C19	0.0721 (16)	0.106 (2)	0.101 (2)	0.0296 (15)	0.0072 (15)	−0.0133 (16)
C20	0.0701 (14)	0.0927 (16)	0.0630 (14)	0.0226 (13)	0.0031 (11)	−0.0155 (12)

### Geometric parameters (Å, °)

**Table:**

F1—C16	1.354 (3)	C8—C9	1.412 (2)
N1—C9	1.378 (3)	C9—C10	1.387 (3)
N1—C14	1.454 (3)	C10—C11	1.362 (3)
N1—H1A	0.8600	C10—H10A	0.93
N2—C1	1.362 (2)	C11—C12	1.376 (3)
N2—C7	1.391 (2)	C11—H11A	0.93
N2—C14	1.451 (2)	C12—C13	1.368 (3)
N3—C1	1.311 (2)	C12—H12A	0.93
N3—C2	1.398 (3)	C13—H13A	0.93
C1—C8	1.439 (3)	C14—C15	1.514 (3)
C2—C7	1.387 (3)	C14—H14A	0.98
C2—C3	1.388 (3)	C15—C16	1.367 (3)
C3—C4	1.372 (3)	C15—C20	1.382 (3)
C3—H3B	0.93	C16—C17	1.369 (3)
C4—C5	1.394 (3)	C17—C18	1.352 (4)
C4—H4A	0.93	C17—H17A	0.93
C5—C6	1.378 (3)	C18—C19	1.370 (4)
C5—H5A	0.93	C18—H18A	0.93
C6—C7	1.377 (3)	C19—C20	1.380 (3)
C6—H6A	0.93	C19—H19A	0.93
C8—C13	1.400 (3)	C20—H20A	0.93
			
C9—N1—C14	122.21 (14)	C11—C10—H10A	119.6
C9—N1—H1A	118.9	C9—C10—H10A	119.6
C14—N1—H1A	118.9	C10—C11—C12	121.1 (2)
C1—N2—C7	106.81 (14)	C10—C11—H11A	119.4
C1—N2—C14	126.12 (16)	C12—C11—H11A	119.4
C7—N2—C14	126.01 (16)	C13—C12—C11	119.9 (2)
C1—N3—C2	104.34 (15)	C13—C12—H12A	120.1
N3—C1—N2	113.37 (17)	C11—C12—H12A	120.1
N3—C1—C8	128.24 (17)	C12—C13—C8	120.18 (19)
N2—C1—C8	118.36 (15)	C12—C13—H13A	119.9
C7—C2—C3	119.58 (19)	C8—C13—H13A	119.9
C7—C2—N3	110.58 (16)	N2—C14—N1	107.87 (15)
C3—C2—N3	129.82 (18)	N2—C14—C15	111.05 (14)
C4—C3—C2	118.3 (2)	N1—C14—C15	112.81 (16)
C4—C3—H3B	120.8	N2—C14—H14A	108.3
C2—C3—H3B	120.8	N1—C14—H14A	108.3
C3—C4—C5	121.1 (2)	C15—C14—H14A	108.3
C3—C4—H4A	119.4	C16—C15—C20	116.13 (19)
C5—C4—H4A	119.4	C16—C15—C14	121.23 (17)
C6—C5—C4	121.4 (2)	C20—C15—C14	122.60 (17)
C6—C5—H5A	119.3	F1—C16—C15	117.59 (19)
C4—C5—H5A	119.3	F1—C16—C17	118.3 (2)
C7—C6—C5	116.7 (2)	C15—C16—C17	124.1 (2)
C7—C6—H6A	121.6	C18—C17—C16	118.2 (2)
C5—C6—H6A	121.6	C18—C17—H17A	120.9
C6—C7—C2	122.92 (19)	C16—C17—H17A	120.9
C6—C7—N2	132.26 (18)	C17—C18—C19	120.6 (2)
C2—C7—N2	104.81 (17)	C17—C18—H18A	119.7
C13—C8—C9	119.59 (19)	C19—C18—H18A	119.7
C13—C8—C1	122.78 (16)	C18—C19—C20	119.9 (3)
C9—C8—C1	117.62 (17)	C18—C19—H19A	120.1
N1—C9—C10	121.90 (17)	C20—C19—H19A	120.1
N1—C9—C8	119.68 (18)	C19—C20—C15	121.1 (2)
C10—C9—C8	118.33 (19)	C19—C20—H20A	119.5
C11—C10—C9	120.87 (19)	C15—C20—H20A	119.5
			
C2—N3—C1—N2	−2.4 (2)	C13—C8—C9—C10	−0.8 (3)
C2—N3—C1—C8	176.00 (18)	C1—C8—C9—C10	178.52 (16)
C7—N2—C1—N3	3.0 (2)	N1—C9—C10—C11	−174.8 (2)
C14—N2—C1—N3	171.79 (16)	C8—C9—C10—C11	1.8 (3)
C7—N2—C1—C8	−175.55 (15)	C9—C10—C11—C12	−1.2 (4)
C14—N2—C1—C8	−6.8 (3)	C10—C11—C12—C13	−0.5 (4)
C1—N3—C2—C7	0.9 (2)	C11—C12—C13—C8	1.4 (3)
C1—N3—C2—C3	−177.1 (2)	C9—C8—C13—C12	−0.8 (3)
C7—C2—C3—C4	−0.5 (3)	C1—C8—C13—C12	179.9 (2)
N3—C2—C3—C4	177.4 (2)	C1—N2—C14—N1	24.3 (2)
C2—C3—C4—C5	0.8 (4)	C7—N2—C14—N1	−168.98 (16)
C3—C4—C5—C6	−0.7 (4)	C1—N2—C14—C15	−99.8 (2)
C4—C5—C6—C7	0.4 (3)	C7—N2—C14—C15	66.9 (2)
C5—C6—C7—C2	−0.2 (3)	C9—N1—C14—N2	−33.8 (2)
C5—C6—C7—N2	−178.7 (2)	C9—N1—C14—C15	89.2 (2)
C3—C2—C7—C6	0.2 (3)	N2—C14—C15—C16	−146.77 (19)
N3—C2—C7—C6	−178.02 (19)	N1—C14—C15—C16	92.0 (2)
C3—C2—C7—N2	179.11 (18)	N2—C14—C15—C20	35.6 (3)
N3—C2—C7—N2	0.9 (2)	N1—C14—C15—C20	−85.7 (2)
C1—N2—C7—C6	176.5 (2)	C20—C15—C16—F1	179.1 (2)
C14—N2—C7—C6	7.7 (3)	C14—C15—C16—F1	1.3 (3)
C1—N2—C7—C2	−2.2 (2)	C20—C15—C16—C17	−0.5 (4)
C14—N2—C7—C2	−171.00 (16)	C14—C15—C16—C17	−178.3 (2)
N3—C1—C8—C13	−3.7 (3)	F1—C16—C17—C18	−179.1 (2)
N2—C1—C8—C13	174.62 (18)	C15—C16—C17—C18	0.4 (4)
N3—C1—C8—C9	176.94 (18)	C16—C17—C18—C19	0.2 (4)
N2—C1—C8—C9	−4.7 (3)	C17—C18—C19—C20	−0.7 (5)
C14—N1—C9—C10	−157.02 (18)	C18—C19—C20—C15	0.6 (4)
C14—N1—C9—C8	26.4 (3)	C16—C15—C20—C19	0.0 (4)
C13—C8—C9—N1	175.87 (18)	C14—C15—C20—C19	177.7 (2)
C1—C8—C9—N1	−4.8 (3)		

### Hydrogen-bond geometry (Å, °)

**Table:**

*D*—H···*A*	*D*—H	H···*A*	*D*···*A*	*D*—H···*A*
N1—H1A···N3^i^	0.86	2.31	2.995 (2)	136
C11—H11A···F1^ii^	0.93	2.43	3.264 (3)	149

Symmetry codes: (i) *x*, −*y*+1/2, *z*−1/2; (ii) −*x*+1, *y*+1/2, −*z*+1/2.
